# Effect of an Er,Cr:YSGG Laser on the Surface of Implants: A Descriptive Comparative Study of 3 Different Tips and Pulse Energies

**DOI:** 10.3390/dj8040109

**Published:** 2020-09-30

**Authors:** Ehsan Chegeni, Antonio España-Tost, Rui Figueiredo, Eduard Valmaseda-Castellón, Josep Arnabat-Domínguez

**Affiliations:** Faculty of Medicine and Health Sciences, University of Barcelona, 08907 Barcelona, Spain; ehsanchegeni2010@yahoo.com (E.C.); toniespana@ub.edu (A.E.-T.); ruipfigueiredo@hotmail.com (R.F.); eduardvalmaseda@ub.edu (E.V.-C.)

**Keywords:** Er,Cr:YSGG laser, peri-implantitis, peri-implant mucositis, implant surface damage

## Abstract

Peri-implant diseases are one of the main complications of dental implants. There are no well-established guidelines regarding laser parameters for implant decontamination. The aim was to compare two different settings of irradiation of the Er,Cr:YSGG laser on dental implants regarding surface alterations and determine the best settings for less damage on the surface. An in vitro study was performed and 30 areas of dental implants were irradiated with two different regimes of energy per pulse 50 and 84 mJ (1.5 W/30 Hz and 2.5 W/30 Hz). A total of 30 sites of implants were irradiated with three different tips (10 surfaces per tip): conical (RTF3-17 mm), side firing (SFT8-18 mm) and cylindrical (MGG6-6 mm). The following descriptive classification on surface damage was employed: no damage (class A), minimal effects (class B), metal fall with melting (class C), and destruction with carbonization (class D). The assessment was made through a descriptive scanning electron microscope (SEM) analysis. Side firing and conical tips at 50 mJ were classified as class A. Side firing at 84 mJ and cylindrical tips 50 mJ and 84 mJ were classified as class B. Finally, class C defects were found in the areas where the conical tip was used at 84 mJ. Side firing and conical tips at 50 mJ do not seem to damage the implant surface.

## 1. Introduction

Oral rehabilitation using implants is a highly documented treatment in scientific literature and has a high success rate with more than 10 years of follow-up [1]. Despite its high success rate, implant treatment can have both mechanical and biological complications [2]. Peri-implantitis is a plaque-associated pathological condition occurring in tissues around dental implants, characterized by inflammation in the peri-implant mucosa and subsequent progressive loss of supporting bone [3]. According to a systematic review and meta-analysis of 15 articles with a long follow-up, mucositis affects approximately 50% of patients and peri-implantitis around 21% [4]. Depending on the sample features and diagnostic criteria, these figures can be significantly higher, and peri-implantitis can affect almost half of the patients [5].

According to two papers by Cortés-Acha et al. [6,7], implants exposed to the oral cavity are extensively covered by biofilm formed by a diverse microbiota. Thus, the treatment of peri-implantitis should include decontamination of the implant surface that has experienced bone loss. Several methods have been described to remove the biofilm attached to the implant (chemical, physical and combination of both) [8]. The decontamination of the implant surfaces with a low-energy Er:YAG laser also appears to have positive outcomes [9].

A recent ex vivo study concluded that laser irradiation of titanium implant surfaces using a 9.3 mm carbon dioxide laser with an average power of 0.7 W showed no increase in temperature of the implant body and surrounding tissues, as well as no evidence of implant surface damage [10]. Although removal of the biofilm without damaging the implant surface is one of the main aims of laser therapy for peri-implant diseases, laser tips, settings and points of application are still a matter of clinical debate.

Therefore, the aim of this study was to compare two different settings of irradiation of the Er,Cr:YSGG laser on implants regarding surface alterations and to determine which application tip of the above-mentioned laser provides less damage to the dental implant surface.

## 2. Materials and Methods

A Waterlase iPlus laser, a class IV Er,Cr:YSGG laser with 2780 nm wavelength (Biolase^®^, Irvine, CA, USA), was used in 15 Avinent^®^ Coral HE implants (AVINENT, Santpedor, Spain). The implants had a diameter of 3.3 mm, were 10 mm long and had a surface sandblasted by alumina and oxidized with calcium and phosphorus.

Using a carborundum disk, a groove was made to divide the implant into 2 sides, so different treatments could be applied. Then, the implants were cleaned with a non-abrasive air spray.

### 2.1. Randomization of Samples

The website www.randomization.com was employed to allocate each side of the implant to a different laser setting. The laser was used for 60 s with 40% water and 50% air at 30 Hz and 1.5 W (50 mJ per pulse) on one side, as recommended by the manufacturer to treat the peri-implantitis, and 2.5 W (84 mJ per pulse) on the other side (with the same frequency, water and air settings). Three different tips were used. Thus, there were 3 subgroups of 5 implants treated with the 3 different tips (Conical tip of 415 μm (RTF3-17 mm), the side firing tip of 800 μm (SFT8-18 mm), and the cylindrical tip of 600 μm (MGG6-6 mm)), each implant being lased on opposite sides with 1.5 or 2.5 W/30 Hz.

### 2.2. Irradiation of the Implants

The irradiation of the implants was carried out to replicate a clinical scenario of peri-implant diseases treatment. The side firing and the conical tips were kept parallel and in contact with the surface of the implant, and continuous vertical movements were made from top to bottom (lateral movements were avoided), with a 2 mm per second speed. The irradiated area was of approximately 2 squared cm. In this way, the application resembles what occurs in patients with peri-implant diseases without opening a surgical flap.

**Figure 1:**
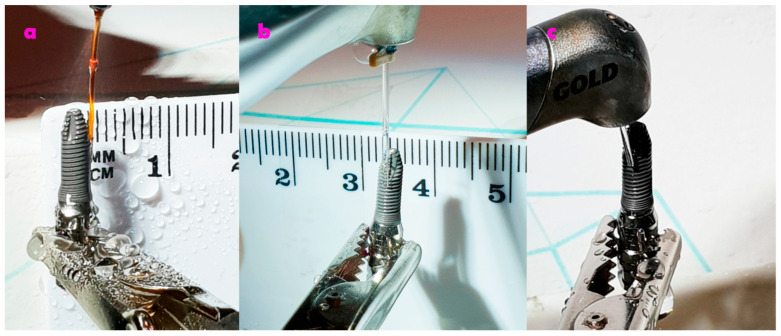
Protocol of irradiation. (**a**) Conical type of 415 micrometer (RTF3-17 mm) tip; (**b**) 800 micrometer side firing tip (SFT8-18 mm); (**c**) 600 micrometer cylindrical tip (MGG6-6 mm).

On the other hand, the cylindrical tip was applied perpendicular and oblique to the implant surface, thus mimicking the situation when a surgical flap is raised (Figure 1), The irradiated area was of approximately 2 squared cm. Table 1 describes the dosimetry of the Er,Cr:YSGG laser.

### 2.3. Scanning Electron Microscope (SEM) Analysis

A gold bath on the implant surfaces using diode sputtering (Diode JFC-100 JEOL Coater; JEOL USA, Inc., Peabody, MA, USA) was deposed. After sputtering, the implants were placed, one by one, in a Quanta 200 SEM (FEI Co., Hillsboro, OR, USA) in an insulating cavity at a 55 degrees angle. The xT Microscope Control Software (FEI Co., Hillsboro, OR, USA) was employed to process the digital images. A chemical analysis of the implants was performed using the EDAX Genesis program (AMETEK Materials Analysis Division, Mahwah, NJ, USA).

The alterations of the implant surface were assessed and classified according to the following criteria:

Class A: No visible damage.

Class B: Minimal effects (areas with small marks without any metal fall or carbonization).

Class C: Metal fall with melting of the surface, considerable fusion and crystallization, visible cracks and small crater-like defects.

Class D: Complete alteration of surface including carbonization with large deep craters.

The classification was carried out observing images at different magnifications (28×, 100×, 500×, 1500×, 3000× and 5000×) in each sample and comparing it with a control implant. To reduce the risk of bias when assessing this variable, two blinded researchers, unaware of the employed parameters and tips, analyzed different photos of samples with different magnifications (including the control). In case of discrepancy, a consensus was reach between the researchers.

## 3. Results

**Figure 2:**
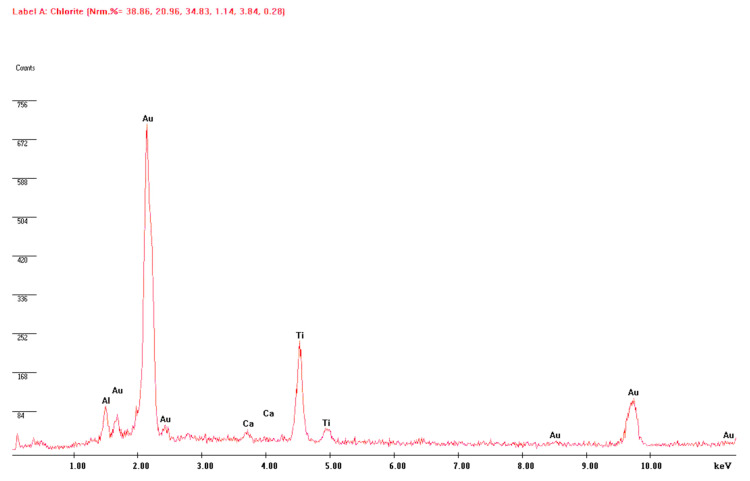
Chemical components of implant. Titanium (Ti), calcium (Ca), aluminum (Al) and gold (Au) were the most commonly detected elements. Some of these are probably related to the coating used to process the samples before the scanning electron microscope (SEM) observation. Kev: Kilo electronvolts; Counts: number of elements presented.

Chemical analysis of the implants disclosed several elements: titanium, calcium, aluminum, gold (coating) and phosphorus. (Figure 2).

**Figure 3:**
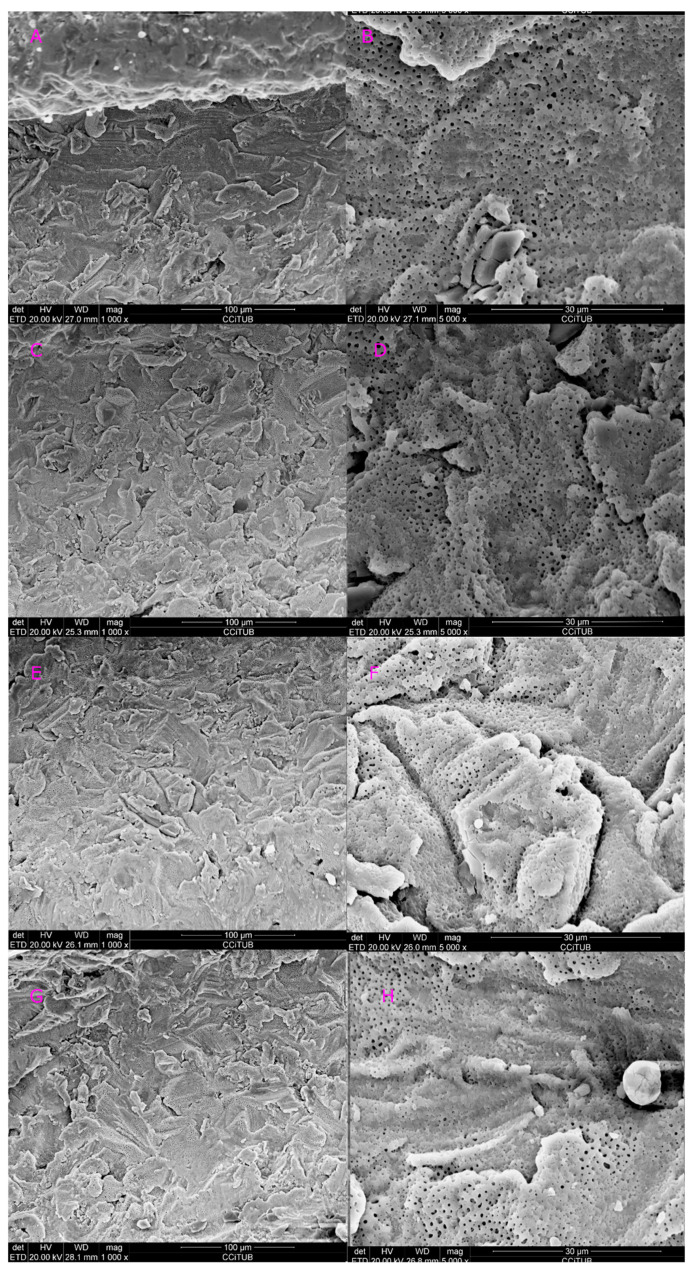
Control versus all 3 tips at 1.5 W. (**A**) Control at 1000× magnification; (**B**) control at 5000× magnification; (**C**) conical tip (RTF3-17 mm) at 1000× magnification; (**D**) conical tip (RTF3-17 mm) at 5000× magnification; (**E**) side firing tip (SFT8-18 mm) at 1000× magnification; (**F**) side firing tip (SFT8-18 mm) at 5000× magnification; (**G**) cylindrical (MGG6-6 mm) tip at 1000× magnification; (**H**) cylindrical (MGG6-6 mm) tip at 5000× magnification.

**Figure 4:**
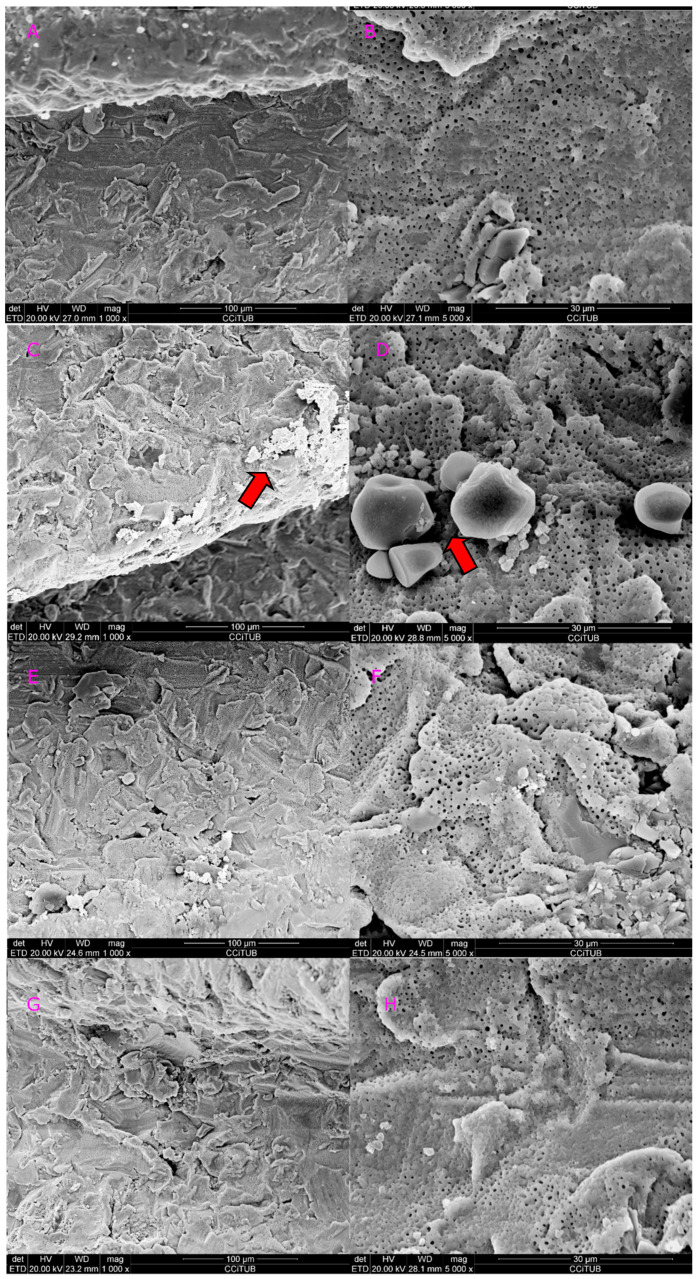
Control versus all 3 tips at 2.5 W. (**A**) Control at 1000× magnification; (**B**) control at 5000× magnification; (**C**) conical (RTF3-17 mm) tip at 1000× magnification; (**D**) conical tip (RTF3-17 mm) at 5000× magnification; (**E**) side firing tip (SFT8-18 mm) at 1000× magnification; (**F**) Side firing tip (SFT8-18 mm) at 5000× magnification; (**G**) cylindrical tip (MGG6-6 mm) at 1000× magnification; (**H**) cylindrical tip (MGG6-6 mm) at 5000× magnification.

Figure 3 and Figure 4 show a comparison of all tips vs. control at 1.5 and 2.5 W. Implants lased with the 415 micrometers conical tip at 1.5 W, showed no signs of surface alterations (class A; Figure 3C,D). However, when the power was increased to 2.5 W, some metal alteration, fusion and crystallization of surface were observed (class C; Figure 4C,D). When the 800 micrometers side firing lateral tip at 1.5 W, no surface damage was observed (class A; Figure 3E,F). Nevertheless, when the power was increased (2.5 W), minimal fusion and crystallization was observed (class B; Figure 4E,F). Finally, implants lased with the 600 micrometers cylindrical tip at 1.5 and 2.5 W induced a reduction in the surface roughness, without any metal fall (class B; Figure 3G,H and Figure 4G,H). Table 2 shows the main study outcomes.

## 4. Discussion

There is currently a great discussion about the best way to decontaminate the surface of implants in the treatment of peri-implantitis. The use of local or systemic medications, laser application, mechanical and/or chemical decontamination, and implantoplasty have been suggested in previous reports [11,12,13]. Another important aim should be the reduction of the surface roughness of the implant, since a smooth surface hampers biofilm adhesion. According to the SEM images obtained in the present study, laser irradiation might be a useful tool to reduce surface roughness. However, future studies should analyze if these changes inhibit bacterial growth over the dental implants. Implantoplasty can be a valid alternative to remove biofilm of dental implants and to reduce its roughness [14]. Nevertheless, this approach is far more aggressive than laser irradiation, although it does not seem to reduce fracture resistance of the implants [15].

Although a randomized clinical trial concluded that Er:YAG treatments might reduce bleeding on probing around implants in comparison with subgingival debridement alone [16], the available evidence on the effectiveness of lasers for implant debridement is still very scarce [17]. In case of decontaminating the implant surface by means of laser irradiation, it is necessary to establish which parameters should be employed. Damage to the implant surface and tissues around the implant might occur as a result of lasing, so the settings should be optimized to avoid these complications [18]. Indeed, more studies on the effect of laser on different implant surfaces should be made. The present study employed a descriptive analysis of the SEM images. However, other methods could also be incorporated such as the measurement of the surface roughness.

Park et al. [19] studied machined titanium and anodized discs, under Er,Cr:YSGG and CO2 laser at 1, 2, 3, 4, and 5 W. Both surfaces presented modifications when the power exceeded 3 W. In the present study, conical tips (RTF3-17 mm) at 2.5 W produced greater damage to the implant surfaces. Future research should analyze if these alterations have a clinical impact.

A study [20] evaluated the surface of failed implants; it did not find any bacteria on the coronal portion of implants when they were irradiated with Er,Cr:YSGG laser before their extraction. Another ex vivo study [21] showed that implantoplasty is a superior method for bacteria elimination compared to other methods, such as laser, chemical agents, hydrogen peroxide and airborne-particle abrasion in a failed implant. However, if the objective of decontamination is to not perform any alteration of the implant surface, the use of a diode laser at 3 W is recommended [22].

Regarding of the decontamination methods, a recent study [23] evaluated three methods using sonic scaler, sonic scaler and chemical agent (Perisolv^®^, Zurich, Switzerland) or an Er:YAG laser on three different implant surfaces, machined, sandblasted and acid-etched (SLA) and hydroxyapatite (HA). Er:YAG laser irradiation was reported as the best option for decontamination of the HA-coated implants. The Er,Cr:YSGG laser is more effective in calculus removal and caused less surface roughness compared with citric acid application [24]. Therefore, we can observe that surface type of implants maybe has a correlation with the used method for decontamination.

A study showed that the Er,Cr:YSGG laser with 1 Watt of power, for 30 s, is capable of the complete elimination of A.baumannii and P.aeruginosa biofilm on the implant surface without damaging the surface topography [25]. Regarding the use of the other lasers, such as Nd:YAG, a recent study [26] showed that Q-Switch Nd:YAG laser-assisted biofilm removal has provided a significant elimination of the biofilm on titanium surfaces.

The present study examined different tips mimicking a clinical situation of implant debridement. It seems that the side firing and conical tips using 1.5 W with 40% water and 50% air at 30 Hz during 60 s are less likely to produce alterations to the implant surface. The calculated parameters estimate that the side firing tip (SFT8-18 mm) had less power density in comparison with the other tips, which, in our opinion, is an advantage. Another positive aspect of this tip is that, due to the lateral irradiation, it could be used to introduce the peri-implant sulcus without raising a flap.

More studies are needed to confirm that the Er,Cr:YSGG laser, applied with the above-mentioned parameters and tips, effectively eliminates bacteria. Moreover, it would be interesting to assess whether the surface roughness modification reduces the bacterial growth after laser irradiation. These issues are paramount to determine the value of laser irradiation in the treatment of peri-implant diseases.

## 5. Conclusions

Based on the present findings, an Er,Cr:YSGG laser with side firing and conical tips at 50 mJ (1.5 W/30 Hz), 50% air, 40% water, for 60 s, do not seem to produce implant surface alterations. A higher degree of damage should be expected when the energy per pulse increases to 84 mJ (2.5 W/30 Hz), especially when the conical tips are used.

## Figures and Tables

**Table 1:** Dosimetry of the Er,Cr:YSGG Laser. W: Watts; Hz: Hertz; sec: seconds; µm: micrometers; cm: centimeters; r: radius; d: diameter; A: Area; E_d_: Energy density; J: Joules; E_p_: Pulse energy; P_m_: Mean power.

Entered Parameters				Calculated Parameters			
Power (W)	Frequency (Hz)	Time (s)	Tip and Diameter (µm)	Tip Radius (cm)	Spot Area (cm^2^)	Energy Density	Pulse Energy (J)
				r = d/2/10,000	A = π r^2^	E_d_ = E/A; J/cm^2^	E_p_ = P_m_/*f*
1.5	30	60	Conical (415)	0.02	0.0012	41.66	0.05
2.5	30	60	Conical (415)	0.02	0.0012	66.66	0.08
1.5	30	60	Cylindrical (600)	0.03	0.0028	17.85	0.05
2.5	30	60	Cylindrical (600)	0.03	0.0028	28.57	0.08
1.5	30	60	Lateral (800)	0.04	0.0050	10	0.05
2.5	30	60	Lateral (800)	0.04	0.0050	16	0.08

**Table 2:** Main outcomes of the study. W: Watts; Hz: Hertz; sec: seconds; µm: micrometers; cm: centimeters; r: radius; d: diameter; A: Area; P_d_: Power density; J: Joules; E_p_: Pulse energy; P_m_: Mean power.

Entered Parameters				Surface Alterations Classification
Power (W)	Frequency (Hz)	Time (s)	Tip and Diameter (µm)	
1.5	30	60	Conical (415)	Class A
2.5	30	60	Conical (415)	Class C
1.5	30	60	Cylindrical (600)	Class B
2.5	30	60	Cylindrical (600)	Class B
1.5	30	60	Lateral (800)	Class A
2.5	30	60	Lateral (800)	Class B
